# Therapist self-report reliability and accuracy in individual vs. conjoint systemic family therapy sessions: implications for clinical monitoring

**DOI:** 10.3389/fpsyg.2026.1811889

**Published:** 2026-09-23

**Authors:** Magdalena Marino, Nicole Porter, Molly Bobek, Sean Dunnsue, Aaron Hogue

**Affiliations:** 1 NYC H+H| Roberto Clemente Center, New York, NY, United States; 2 Clinical Psychology Department, Universidad Complutense de Madrid, Madrid, Spain; 3 Partnership to End Addiction, New York, NY, United States

**Keywords:** adolescent therapy, clinical monitoring, session composition, systemic family therapy, therapist self-report

## Abstract

**Purpose:**

This study investigated how session composition (individual vs. conjoint) influenced the reliability and accuracy of therapist self-report on the use of Systemic Family Therapy (SFT) techniques in a community-based mental health clinic (CMHC). Accurate self-reporting is essential for monitoring and improving clinical practice, ensuring treatment fidelity, and evaluating evidence-based models.

**Method:**

Eighteen therapists from a CMHC in New York City submitted 165 session recordings with corresponding self-report scores on use of SFT interventions. Therapist reliability with observational scores was analyzed using intraclass correlation coefficients (ICCs), and accuracy was evaluated through comparisons with observer ratings using linear mixed models. The effect of session composition on extensiveness of SFT technique delivery was tested with a linear mixed model.

**Results:**

Therapists demonstrated strong reliability in reporting SFT techniques overall (ICC = 0.76) and in both individual (ICC = 0.71) and conjoint sessions (ICC = 0.67). However, accuracy varied by session type; therapists tended to overestimate their use of SFT techniques in individual sessions and were more accurate in conjoint sessions. Conjoint sessions also tended to feature a more extensive application of SFT techniques.

**Conclusion:**

These findings suggest that session composition influences the accuracy of therapist self-report and the extensiveness of SFT delivery. The overestimation in individual sessions highlights the need for enhanced training and supervision to improve ability to accurately report on use of SFT techniques. Further research is needed to explore the factors influencing differences in reporting accuracy between types of sessions and to refine methods for assessing SFT implementation.

## Introduction

In community-based mental health clinics (CMHCs), novice clinicians provide services under the supervision of more experienced clinical staff. For supervisors to successfully monitor and improve therapists’ clinical practice, therapists must be able to recognize and accurately describe the interventions they are implementing. Moreover, successful implementation of evidence-based models in CMHCs depends heavily on therapists’ ability to deliver techniques as intended; otherwise, it becomes impossible to reliably assess the effectiveness of the treatment model (Perepletchikova, 2011; Busse et al., 2021). Therefore, it is essential for the sustainability of evidence-based treatments in CMHCs to better understand therapists’ ability to accurately evaluate and report on what they are doing in their clinical sessions. This need is especially critical in clinics that use systemic family therapy (SFT), where interventions are often multicomponent, relationally oriented, and embedded in real time family interactions—making accurate self-reporting both more challenging and more essential to maintaining fidelity.

Clinical supervision is the first line for quality assurance in treatment, and so improving supervision is a potent and sustainable implementation strategy to support clinicians delivering evidence-based treatment in CMHCs (Chapman et al., 2022; Dorsey et al., 2018). Supervisors and clinical directors in CMHCs rely on therapists to adhere to evidence-based treatment models and deliver techniques as expected. One way supervisors and clinical directors monitor treatment delivery is via therapist self-report on intervention delivery during clinical supervision. For this to be effective, therapist self-report needs to capture three complementary aspects of intervention delivery: **reliability,** referring to the consistency with which therapists report their use of techniques across raters; **accuracy**, referring to the agreement in magnitude between therapist self-report and independent observer ratings of the same session; and **extensiveness**, referring to the frequency and thoroughness with which a technique is delivered during a session (Hogue et al., 2015). Although therapist self-report is widely used in clinical supervision, research shows that they are often misaligned with ratings from independent observers with notable limitations in accuracy and reliability (Brookman-Frazee et al., 2021). This reflects a long-standing pattern in therapist–observer concordance studies (see McLeod et al., 2023); previous studies have found modest to weak alignment between therapist self-report and observer ratings of the delivery of treatment models and techniques in community health settings (Xenakis et al., 1983; Carroll et al., 1998; Ward et al., 2013; Peavy et al., 2014; Sheshko et al., 2020; Herschell et al., 2020; Beckman et al., 2022; Harned et al., 2023), with therapists tending to overestimate the extent of their evidence-base treatment use (Martino et al., 2009; Hurlburt et al., 2010; Chapman et al., 2013; Borntrager et al., 2015; Brookman-Frazee et al., 2021).

Research examining therapist self-report in systemic and family therapy has converged on three general findings. First, therapists typically demonstrate moderate to high agreement with independent observers regarding whether core SFT techniques are implemented during routine clinical practice (Busse et al., 2021; Hogue and Dauber, 2013; Hogue et al., 2015, 2021, 2022). Second, despite generally acceptable reliability, therapists frequently overestimate the extensiveness with which they deliver these interventions, suggesting that consistent identification of techniques does not necessarily translate into accurate judgments about their frequency or thoroughness (Chapman et al., 2013; Hogue et al., 2015; Hurlburt et al., 2010; Martino et al., 2009). Third, therapist self-report appears to be influenced by characteristics of the treatment context. Reliability tends to be stronger in highly structured or manualized interventions, particularly during earlier phases of treatment, and weaker as treatment becomes more flexible and individualized (Brookman-Frazee et al., 2021; Couturier et al., 2021).

Together, these results suggest that family therapists can provide reliable self-report on their use of Family Therapy techniques during routine sessions, though they may be weaker on accuracy. Further, the contextual factors influencing its reliability and accuracy remain poorly understood. The question remains why SFT interventions are self-rated with greater reliability than other structured interventions. One contextual factor that may explain these findings is session composition. Some SFT techniques can only be implemented—or are more readily observable—when multiple family members are present, potentially improving both therapists’ recognition of their own interventions and observers’ ability to identify them (Hogue et al., 2015). Similarly, when multiple individuals are present, therapists may use certain SFT techniques more thoroughly or frequently (i.e., more extensively). Brookman-Frazee et al. (2021) found that the extensiveness of use of a specific therapy technique can improve reliability when therapists are able to carry out planned activities and when the therapy is highly structured. However, the presence of multiple persons in the room may also create an exaggerated impression of systemic engagement, even if actual interventions remain individually focused or linear (Nichols and Tafuri, 2013), leading therapists to overestimate their use of SFT techniques, as has been reported in previous studies (Hurlburt et al., 2010; Hogue et al., 2015, 2021). Despite these theoretical possibilities, the role of session composition in shaping therapist self-report has not been examined directly.

While SFT is more frequently conducted in multi-person sessions, it can also be practiced in individual sessions when therapists maintain a focus on the client’s relational and contextual systems (Szapocznik et al., 1990; McGoldrick and Carter, 2001; Moreno, 2018). Grounded in systems and communication theory (Bateson, 1972), SFT emphasizes the influence of the relational context on mental health, conceptualizing clients’ difficulties within the broader context of their relationships, family dynamics, internalized family roles, and social systems, rather than viewing problems as solely intrapsychic or individual. SFT can be delivered effectively in both conjoint and individual sessions by ensuring that assessment, formulation, and intervention are always anchored in an understanding of clients as part of interconnected relational and systemic patterns, and using systemic principles such as circular questioning, genograms, and reframing (Russell and Breunlin, 2019; Sheinberg and Brewster, 2014). Practical challenges associated with convening entire families in real-world clinical settings, along with the increasing need for integrative models that can be tailored to an evolving society, have contributed to the development of more flexible approaches within SFT practice (Breunlin and Jacobsen, 2014). It is now widely accepted that SFT can be successfully delivered to whole families, subsystems, or individual clients across a broad range of clinical concerns and populations, while remaining grounded in systemic processes (Moreno, 2018; Todd et al., 2025; Russell and Breunlin, 2019; Lebow, 2019). The flexibility with which SFT can be utilized, in a variety of session compositions and with a wide range of problems and populations, makes it an ideal modality for researching the factors that shape the reliability and accuracy with which therapists report on their use of techniques.

## The current study

The current study examined the effect of session composition (conjoint versus individual) on therapist self-report accuracy and reliability on their use of SFT core techniques in routine sessions with adolescents and families in a CMHC. The clinic does not use manualized SFT models. Instead, the clinic provides extensive training on the Ecological-Systemic Family Therapy approach (Inclán, 2009; Inclan and Mariño, 2022). Drawing from Bronfenbrenner’s ecological framework, the Ecological-Systemic Model conceptualizes mental health within interacting individual, family, community, and sociocultural systems, emphasizing relational processes while incorporating broader ecological influences into assessment and intervention. The specific interventions assessed include seven core SFT techniques distilled from Functional Family Therapy (FFT; Sexton and Alexander, 2003), Multidimensional Family Therapy (MDFT; Liddle, 2010), and Brief Strategic Family Therapy (BSFT; Szapocznik et al., 2012), three evidence-based family therapy models for adolescent behavioral and substance use problems (Baldwin et al., 2012; Hogue et al., 2019). Interventions consisted of targeting family communication and attachment, parental monitoring and family rules, live coaching of family interactions, preparation for conjoint sessions, normative adolescent development, targeted work with participating family members, and core relational family themes (see Table 1). These techniques represent common systemic practice elements shared across evidence-based family therapy models rather than adherence to any single treatment manual. These core techniques are transdiagnostic systemic elements, targeting shared underlying mechanisms across multiple disorders rather than being designed for a single diagnosis, grounded in the SFT theoretical framework, but not bound to a specific protocol or disorder-specific manuals (Marchette and Weisz, 2017). Because of that, they can be more flexibly implemented across a diverse range of clinical presentations, settings, and patient needs (Hogue et al., 2019), providing an opportunity to examine whether the relational context in which therapy occurs—specifically, individual versus conjoint sessions—influences both the delivery of systemic interventions and therapists’ ability to recognize and accurately report their own practice.

**Table 1:** Core systemic family therapy techniques assessed in the ITT-ABP family therapy subscale.

Core techniques	Description
Parental monitoring and family rules	Interventions that strengthen parental monitoring, family rules, caregiving responsibilities, and generational boundaries to improve family structure and adolescent behavioral regulation.
Prepared for family session	Individual or subsystem work that prepares adolescents or caregivers for future family interactions by anticipating reactions, reducing resistance, and promoting more productive relational exchanges.
Arranged and coached family interactions	Therapist facilitates live interactions between family members, coaches communication during the exchange, and processes the interaction to promote new relational patterns.
Family attachment and communication	Interventions that strengthen communication, emotional connection, and attachment by improving interactional patterns, perspective-taking, and mutual understanding among family members.
Normative adolescent development	Psychoeducation that reframes adolescent behavior within normative developmental processes to promote realistic expectations and more adaptive family responses.
Targeted family members for change	Direct interventions aimed at helping a participating family member modify thoughts, emotions, or behaviors that contribute to relational difficulties within the family system.
Core relational family themes	Exploration of underlying relational themes (e.g., trust, respect, autonomy, loyalty, fairness) that organize recurring family interactions and give meaning to everyday conflicts.

This study has two main aims: (1) testing how session composition impacts reliability and accuracy on therapists’ self-report of SFT technique use, and (2) determining how session composition affects the extensiveness of intervention delivery. By examining these questions in routine, non-manualized community-based care, the study contributes to understanding how systemic interventions are enacted in community settings and provides evidence that can inform training and supervision, ultimately contributing to improvements in treatment quality.

To address these gaps, this study will compare therapist self-report with observer ratings in conjoint and individual sessions to determine (a) the overall reliability and accuracy of therapist self-report, (b) whether reliability and accuracy vary by session type, and (c) whether the extensiveness of SFT technique delivery differs between conjoint and individual sessions. Based on prior research, we hypothesized that (1) therapist self-report would demonstrate strong reliability relative to observer ratings across both session types; (2) therapist self-report would be less accurate in conjoint than in individual sessions; and (3) therapists would tend to overestimate their use of SFT techniques relative to observer ratings across both session types.

## Method

### Participants

The present study is a secondary analysis of an observational dataset originally collected as part of a larger investigation of therapist fidelity in community-based family therapy using therapist self-report and observational coding. The current analyses address a novel research question—whether session composition influences therapist self-report reliability, accuracy, and extensiveness of systemic family therapy (SFT) technique delivery—that has not previously been examined using these data (Hogue et al., 2017).

The dataset comprised 165 therapy session recordings collected from 18 therapists at a CMHC in New York City between November 2007 and July 2012. Treatment sessions were based on the Ecological Systemic Model (ESM; Inclán, 2009; Inclan and Mariño, 2022) (see above). Because the study examines therapist and observer ratings of core systemic intervention techniques rather than disorder-specific treatment protocols, the research question is independent of changes in disorder specific treatment protocols over time.

We used non-randomized convenience sampling. All therapists at the clinic were invited to participate voluntarily. Therapists were psychologists (*n* = 3), clinical social workers (*n* = 11), and doctoral-level psychology interns with experience in SFT (*n* = 4). Before participating in the study, all therapists completed a 3-h training session on the ITT-ABP Family Therapy techniques and scoring procedures. Throughout the study, therapists continued to participate in the clinic’s routine supervision and training in ESM. Participating therapists ranged from 23 to 59 years old (*M* = 31.7 years, SD = 9.4), all were bilingual (Spanish/English) and cisgender; 15 were female, 14 were Hispanic American, and as a group they averaged 3.8 years (SD = 3.9) post-graduate therapy experience.

The dataset included therapy sessions from 104 adolescents receiving routine outpatient services at the clinic. Adolescents ranged in age from 13 to 18 years (*M* = 15.61 years). The sample was predominantly Hispanic (64%), and 68% lived in single-parent households. The most common presenting diagnoses were Oppositional Defiant Disorder (82%) and Attention-Deficit/Hyperactivity Disorder (68%), and 74% of adolescents met criteria for more than one psychiatric diagnosis.

### Instruments

This study used the Inventory of Therapy Techniques for Adolescent Behavior Problems (ITT-ABP) as well as its observational version (ITT-ABP-O) (Hogue et al., 2012). The ITT-ABP is a post-session therapist-report measure of 27 therapeutic techniques representing four theoretical approaches to treating adolescents in outpatient behavioral care: Cognitive Behavioral Therapy, SFT, Motivational Interviewing, and Drug Counseling (Hogue et al., 2012). The ITT-ABP has demonstrated factor validity, discriminant validity, and convergent validity (Hogue et al., 2014). The observational version (ITT-ABP-O), which contains identical Family Therapy items rated by trained observers, has also demonstrated adequate psychometric properties, including good inter-observer reliability and therapist-observer agreement (Hogue et al., 2015). In the present sample, internal consistency for the SFT subscale was acceptable for both therapist ratings (Cronbach’s *α* = 0.72) and observer ratings (Cronbach’s *α* = 0.73).

This study used the seven techniques included in the SFT subscale: parental monitoring and family rules, preparation for family sessions, arranged and coached family interactions, family attachment and communication, normative adolescent development, targeted work with participating family members, and core relational family themes (see Table 1). Therapists rated these seven items on a five-point Likert scale [0 = *Not at all* to 4 = *Extensively*] to indicate the thoroughness and frequency with which they utilized each technique in a recently completed session.

### Procedures

Study therapists submitted audio or video recordings of therapeutic sessions with adolescents (ages 13–18) and/or their caregivers. Families were informed of the research goals and invited to participate in the study; interested families signed a written consent form upon agreeing to participate. During each visit, therapists recorded an audio or video tape of the entire session. Immediately following the session, they completed the ITT-ABP based on the session they had just concluded. Therapists and families were informed that they could stop the recording or withdraw from the study at any time. During the study, therapists were asked to submit session recordings and corresponding self-report for as many sessions as possible.

Observational coders were research personnel trained to code the delivery of SFT techniques. The rater cohort included undergraduates and graduates with a bachelor’s or master’s level training in psychology or a related field. The group consisted of 5 European American women, 2 European American men, 1 African American woman, 1 Hispanic American woman, and 1 Asian American woman. None had prior fidelity coding experience. Observational coders used the ITT-ABP-O to rate the extensiveness with which therapists delivered interventions during recorded sessions. Raters completed a structured two-month training program consisting of twice-weekly meetings that included review of the rating manual, operational definitions of intervention techniques, and in-group coding exercises. Study coding began once the rating cohort reached a collective reliability threshold of ICC = 0.65 (Shrout and Fleiss, 1979) for 80% of items. Throughout the study, the coding cohort met weekly for booster training and rater drift monitoring until study coding was complete. Each study session was scored by two randomly paired observers.

### Statistical analysis

Statistical analyses were performed using SPSS Statistics 27.0. Descriptive statistics and model diagnostics were examined to evaluate distributions, outliers, and relevant model assumptions. We conducted a preliminary analysis to examine interrater reliability by calculating the ICC for the two observer scores for the full scale (SFT Total Score) and each item. Once the interrater reliability was established, we averaged observer scores to be used as the observational score in all further analyses.

To examine therapist reliability on reporting SFT techniques, we calculated therapist-observer reliability using one-way random ICCs for SFT Total Score, and for each of the items. We followed Cicchetti’s criteria (Cicchetti, 1994) to define the magnitude of the ICCs, so that values below 0.40 reflected “Poor” agreement, 0.40 to 0.59 “Fair” agreement, 0.60 to 0.74 “Good” agreement, and 0.75 and higher “Excellent” agreement. To assess the effect of session composition on therapist reliability, we calculated the ICCs separately for conjoint and individual sessions.

To assess the accuracy of therapist self-report scores we used linear mixed modeling comparing therapist and observer ratings on each technique and SFT Total score. Because multiple therapy sessions were available for some families, **client** was specified as a random intercept in all models to account for the non-independence of repeated observations. Rater (therapist vs. observer) was entered as a fixed effect. Effect sizes were calculated using partial eta squared (η2) and were classified as small (η2 = 0.01), medium (η2 = 0.06), and large (η2 = 0.14) following the conventional classification (Pallant, 2020). The effect of session composition on therapist accuracy was examined with a similar linear mixed-model analysis adding session composition (individual, conjoint) as a fixed effect and analyzing the interaction between rater and session composition.

To assess the effect of session composition on extensiveness of SFT technique delivery, we utilized a similar linear mixed-model approach. Using observer report data, we tested SFT Total Score and each technique. In these models, client was classified as a random effect and session composition as a fixed effect.

## Results

The 165 therapy session recordings represented 104 families. Ninety-eight (59%) were individual sessions and 67 (41%) were conjoint sessions. Preliminary analyses of inter-observer reliability using one-way random ICCs indicated excellent agreement for the SFT Total Score (ICC = 0.88), and good to excellent agreement across the seven individual items that represent the SFT Scale (ICC range = 0.63–0.88; Cicchetti, 1994). These finding justified averaging observer ratings to create a single observational score for subsequent analyses.

### Therapist self-report reliability

Intraclass correlation coefficients for the full sample and stratified by session composition are presented in Table 2. Results indicated excellent therapist-observer reliability for the SFT Total Score in the full sample (ICC = 0.76), and good agreement for both individual (ICCind = 0.71) and conjoint sessions (ICCcon = 0.67). When considering individual items, reliability was good for *Parental monitoring* overall (ICC = 0.72) as well as in individual (ICCind = 0.73) and in conjoint (ICCcon = 0.61) sessions. In contrast, reliability was poor for *Core relational family themes* both overall (ICC = 0.31) and for each session composition (ICC ind = 0.26; ICC con = 0.24). For all other items, agreement ranged mainly between fair and poor, with some substantial differences between session compositions. The item with the greatest difference in agreement between session composition was *Prepared for family session*, which showed poor agreement both overall (ICC = 0.26) and for individual sessions (ICCind = 0.10), but good agreement for conjoint sessions (ICCcon = 0.66). The item *Normative adolescent development* showed a similar result, with poor agreement both overall (ICC = 0.30) and for individual sessions (ICCind = 0.13), but with fair agreement in conjoint sessions (ICCcon = 0.47). Conversely, the item *Family attachment and communication* had fair agreement overall (ICC = 0.53) and for individual sessions (ICCind = 0.44), but poor agreement for conjoint sessions (ICCcon = 0.34). Finally, for *Coached Family Interactions*, a technique that can only be used in conjoint sessions, there was fair agreement (ICCcon = 0.41).

**Table 2:** Therapist–observer reliability on SFT total score and SFT items.

SFT scale/item	Full sample (*N* = 149)		Individual sessions (*N* = 90)		Conjoint sessions (*N* = 59)	
	ICC	Level of reliability	ICC	Level of reliability	ICC	Level of reliability
SFT Total Score	0.76	Excellent	0.71	Good	0.67	Good
Parental monitoring	0.72	Good	0.73	Good	0.61	Good
Prepared for family session	0.26	Poor	0.10	Poor	0.66	Good
Coached family interactions	--		--		0.41	Fair
Family attachment and communication	0.53	Fair	0.44	Fair	0.34	Poor
Normative adolescent development	0.30	Poor	0.13	Poor	0.47	Fair
Targeted family members	0.47	Fair	0.40	Fair	0.37	Poor
Core relational family themes	0.31	Poor	0.26	Poor	0.24	Poor

ICC = intraclass correlation coefficient. Reliability was interpreted according to Cicchetti (1994): poor (<0.40), fair (0.40–0.59), good (0.60–0.74), and excellent (≥ 0.75).

Overall, therapist-observer reliability was substantially stronger for the aggregated SFT Total Score than for several individual techniques, indicating that agreement varied across specific intervention types.

### Therapist self-report accuracy

Self-report accuracy results are presented in Table 3; only statistically significant findings are summarized here. For SFT Total Score, there was no significant difference between therapist and observer ratings when considering the full sample and for conjoint sessions; however, this was not the case for individual sessions [*F*(1, 238.37) = 16.19, *p* < 0.001, *ηp^2^* = 0.06], for which therapists overestimated their use of SFT techniques. Considering the individual items in the full sample, therapist and observer ratings did not differ significantly for *Parental monitoring and family rules*. Compared to observers, therapists significantly overestimated their use of three techniques: *Prepared for Family Sessions* [*F*(1, 197.51) = 12.21, *p* < 0.001, *ηp^2^* = 0.06], *Normative adolescent development* [*F*(1, 194.72) = 45.75, *p* < 0.001, *ηp^2^* = 0.19], and *Core relational family themes* [*F*(1, 198.39) = 54.90, *p* < 0.001, *ηp^2^* = 0.22] and underestimated their use of two: *Family communication and attachment* [*F*(1, 201.52) = 12.82, *p* < 0.001, *ηp^2^* = 0.06] and *Targeted family members* [*F*(1, 207.18) = 8.08, *p* = 0.005, *ηp^2^* = 0.04]. The effect sizes of the overestimation were medium to large, and small to medium for the underestimation.

**Table 3:** Results of linear mixed models testing therapist accuracy by session composition.

SFT scale/item	Session composition	Observer mean (SD)	Therapist mean (SD)	*F*	Num df	Den df	*p*	*ηp^2^*
SFT total score	Full *N*	1.96 (0.59)	2.21 (0.72)	2.67	1	195.11	0.104	0.01
	Individual	1.77(0.57)	2.05(0.73)	16.19	1	238.37	**<0.001**	0.06
	Conjoint	2.27(0.49)	2.45(0.64)	1.15	1	242.23	0.285	0.00
Parental monitoring and family rules	Full *N*	2.32 (1.18)	2.55 (1.23)	0.28	1	199.98	0.599	0.00
	Individual	2.06(1.06)	2.18(1.27)	0.42	1	241.26	0.520	0.00
	Conjoint	2.75(1.23)	3.04(1.09)	2.57	1	240.44	0.110	0.01
Prepared for family session	Full *N*	1.20 (0.49)	1.76 (1.19)	12.21	1	197.51	**<0.001**	0.06
	Individual	1.19(0.51)	1.99(1.33)	49.22	1	238.36	**<0.001**	0.17
	Conjoint	1.22(0.46)	1.41(0.84)	1.78	1	238.22	0.183	0.01
Arranged, coached, processed interaction	Full *N*	–	–	–	–	–	–	–
	Individual	–	–	–	–	–	–	–
	Conjoint	2.53(0.93)	2.06(1.14)	3.39	1	200.01	0.067	0.02
Family communication and attachment	Full *N*	3.21 (1.17)	2.62 (1.19)	12.82	1	201.52	**<0.001**	0.06
	Individual	2.90(1.16)	2.19(1.14)	28.10	1	243.99	**<0.001**	0.10
	Conjoint	3.71(1.02)	3.25(0.96)	7.41	1	242.67	**0.007**	0.03
Normative adolescent development	Full *N*	1.54 (0.76)	2.34 (1.19)	45.75	1	194.72	**<0.001**	0.19
	Individual	1.39(0.69)	2.27(1.22)	50.85	1	237.36	**<0.001**	0.17
	Conjoint	1.79(0.79)	2.45(1.16)	17.86	1	236.40	**<0.001**	0.07
Targeted family members	Full *N*	1.75 (1.08)	1.51 (0.93)	8.08	1	207.18	**0.005**	0.04
	Individual	1.50(1.05)	1.22(0.65)	6.42	1	253.04	**0.012**	0.02
	Conjoint	2.15(1.01)	1.93(1.11)	0.00	1	251.46	0.985	0.00
Core relational family themes	Full *N*	1.70 (0.77)	2.66 (1.16)	54.90	1	198.39	**<0.001**	0.22
	Individual	1.54(0.64)	2.39(1.14)	49.34	1	238.77	**<0.001**	0.17
	Conjoint	1.96(0.89)	3.06(1.09)	55.78	1	238.36	**<0.001**	0.19

SFT: Systemic Family Therapy, SD: standard deviation, df: degrees of freedom, Num: Numerator, Den: Denominator. Bold values indicate statistically significant results (*p*<.05).

Session composition substantially altered the pattern of therapist self-report accuracy. In individual sessions, therapists showed a similar pattern to that of the full sample; they were accurate only in reporting their use of *Parental monitoring*. Therapists significantly overestimated their use of *Prepared for Family Sessions* [*F*(1,238.36) = 49.22, *p* < 0.001, *ηp^2^* = 0.17], *Normative adolescent development* [*F*(1, 237.36) = 50.85, *p* < 0.001, *ηp^2^* = 0.17], and *Core relational family themes* [*F*(1, 238.77) = 49.34, *p* < 0.001, *ηp^2^* = 0.17]; conversely, they underestimated their use of *Family communication and attachment* [*F*(1, 243.99) = 28.10, *p* < 0.001, *ηp^2^* = 0.10] and *Targeted family members* [*F*(1,253.4) = 6.42, *p* = 0.012, *ηp^2^* = 0.02]. In conjoint sessions, however, therapist and observer ratings did not differ significantly for four of the seven techniques: *Parental Monitoring*, *Prepared for family session*, *Arranged*, *coached interaction*, *and Targeted family members*. Therapists underestimated *Family Attachment and Communication* [*F*(1, 242.67) = 7.41, *p* = 0.007, *ηp^2^* = 0.03], whereas they overestimated *Normative Adolescent Development* [*F*(1, 236.40) = 17.86, *p* < 0.001, *ηp^2^* = 0.07] and *Core Relational Family Themes* [*F*(1, 238.36) = 55.78, *p* < 0.001, *ηp^2^* = 0.019]. The effects were small in the underestimation, but medium to large for the overestimations.

### Observer report of SFT extensiveness

Extensiveness results are presented in Table 4. According to observer ratings, therapists delivered SFT techniques more extensively during conjoint than individual sessions. This difference was reflected in the SFT Total Score, which increased from an average of 1.77 (SD = 0.57) in individual sessions to 2.27 (SD = 0.49) in conjoint session, representing a statistically significant difference [*F*(1, 311.27) = 28.88, *p* < 0.001, *ηp^2^* = 0.08]. Therapists also demonstrated significantly greater extensiveness in three techniques during conjoint sessions: *Parental monitoring* [*F*(1, 301.73) = 6.30, *p* = 0.013, *ηp^2^* = 0.02], *Family communication and attachment* [*F*(1, 266.49) = 7.83, *p* = 0.006, *ηp^2^* = 0.03], and *Core relational family themes* [*F*(1, 229.99) = 6.29, *p* = 0.013, *ηp^2^* = 0.03]. No significant differences were found for *Prepared for family session*, *Normative adolescent development*, or *Targeted family members*.

**Table 4:** Results of linear mixed models comparing extensiveness of SFT delivery in individual and conjoint sessions.

SFT scale/item	Individual mean (SD)	Conjoint mean (SD)	*F*	Num df	Den df	*p*	*ηp^2^*
SFT total score	1.77(0.57)	2.27(0.49)	28.88	1	311.27	**<0.001**	0.08
Parental monitoring and family rules	2.06(1.18)	2.75(1.23)	6.30	1	301.73	**0.013**	0.02
Prepared for family session	1.19(0.51)	1.22(0.46)	0.35	1	252.18	0.554	0.001
Family communication and attachment	2.90(1.16)	3.71(1.02)	7.83	1	266.49	**0.006**	0.03
Normative adolescent development	1.39(0.69)	1.79(0.79)	1.95	1	305.33	0.164	0.01
Targeted family members	1.50(1.05)	2.15(1.01)	1.80	1	277.78	0.180	0.01
Core relational family themes	1.54(0.64)	1.96(0.89)	6.29	1	229.99	**0.013**	0.03

SFT: Systemic Family Therapy, SD: standard deviation, df: degrees of freedom, Num: Numerator, Den: Denominator. Bold values indicate statistically significant results (*p*<.05).

Observer ratings also indicated that therapists implemented a broader range of SFT techniques during conjoint sessions, using an average of 4.2 different techniques per session compared to 2.4 different techniques per session in individual sessions. Observers identified two or more SFT interventions in 94% of conjoint sessions compared with 60% of the individual sessions. Across both session compositions, *Family communication and attachment* was the most frequently observed intervention, followed by *Parental monitoring and family rules*.

## Discussion

This study aimed to assess the reliability and accuracy of therapist self-report on use of SFT interventions in routine, non-manualized sessions within CMHCs. In addition, this study sought to understand the impact of session composition on reliability and accuracy of therapist self-report, a factor which to our knowledge has yet to be closely examined. Overall, therapists demonstrated good agreement with independent observers regarding their use of SFT techniques in all contexts. However, session composition substantially influenced therapist self-report accuracy and the extensiveness with which SFT techniques were delivered. Therapists were generally accurate when reporting their overall use of SFT techniques in conjoint sessions, whereas they systematically overestimated their use of these techniques during individual sessions. These findings suggest that session composition influences not only how systemic interventions are implemented, but also how accurately therapists recognize and report their own clinical practice. As consequence, session composition can be conceptualized as a contextual characteristic of systemic family therapy rather than simply a descriptive feature of treatment delivery.

Our findings regarding therapist reliability align with previous research showing that family therapists are reliable informants on their own clinical practice (Hogue et al., 2015, 2021; Chapman et al., 2013; Busse et al., 2021; Couturier et al., 2021). Although agreement varied across techniques, therapist-observer reliability for the aggregated SFT Total Score remained strong across both session compositions. This pattern is consistent with classical psychometric theory, whereby composite scores typically demonstrate greater reliability than individual items because aggregation reduces random measurement error (Nunnally and Bernstein, 1994). Thus, while agreement differed for specific techniques, therapists consistently identified their overall level of systemic intervention delivery.

Some previous studies, such as Ward et al. (2013), Couturier et al. (2021), and Brookman-Frazee et al. (2021), indicate that reliability improves significantly when sessions are highly scripted, as is often the case in manualized treatment. Our study, however, shows that therapists can also be reliable in non-scripted and/or non-manualized sessions, which are more reflective and a better fit for real world clinical care. It may be that providing therapists with greater autonomy and flexibility in choosing evidence-based SFT techniques during sessions, without fear of deviating from a prescribed model, will increase - rather than diminish - therapist reliability.

Regarding accuracy, our findings suggest that family therapists are accurate in reporting on their use of SFT techniques overall and in conjoint sessions, but less so in individual sessions, for which they mostly overestimated the extent to which they implemented SFT techniques. These findings confirm that session composition impacts therapists’ precision when self-reporting their use of SFT interventions. However, contrary to our hypothesis, therapists were more accurate in conjoint session, which suggests that the presence of multiple family members may enhance the visibility of systemic interventions, facilitating therapist recognition of their own intervention delivery despite the greater complexity of conjoint sessions.

While many previous studies (e.g., Carroll et al., 1998; Martino et al., 2009; Hurlburt et al., 2010; Chapman et al., 2013; Borntrager et al., 2015; Herschell et al., 2020; Brookman-Frazee et al., 2021) found that therapists overestimated the extent to which they implemented therapeutic interventions, this literature base did not consider the impact of session composition. Our study found therapists to be accurate when reporting the overall extent to which they used of SFT interventions and accurately reported their use of four of the seven SFT techniques when there were multiple family members in the room. Yet, in individual sessions, therapists were less accurate and tended to overreport their use of SFT interventions. The largest effects on these significant over estimations were for the techniques *Core relational family themes* and *Normative adolescent development*. Interestingly, *Family communication and attachment*, the most used technique by therapists in this study and one of the pillars of SFT, was underestimated in both session compositions, even more significantly in conjoint sessions. It may be that therapists viewed this intervention as the overall relational context within which they were working rather than as a discrete intervention and therefore rated themselves lower on this specific technique. These results suggest that therapist self-report is trustworthy when it comes to conjoint work but may exaggerate technique usage for individual sessions. These findings also suggest that the observability of systemic interventions differs across session compositions. In conjoint sessions, relational processes are externalized through direct family interaction, whereas individual sessions often require therapists to work through internalized or anticipated family relationships, making the systemic intent of interventions less visible to independent observers. Consequently, differences between therapist self-report and observational coding may reflect not only reporting bias but also the different ways systemic processes manifest across session formats.

There are at least three possible factors contributing to the difference in reliability and accuracy across session compositions. First, despite instructions to report on only interventions delivered in session and not on clinical intentions, therapists may have scored their sessions according to their broader clinical intentions that are not always directly observable. This bias, described also in a recent review of multi-informant research (McLeod et al., 2023), may be especially relevant for individual sessions in SFT, as many interventions delivered in this context are focused on internalized family systems rather than facilitating direct interaction among family members, making them more difficult for observers to identify. The second factor is that while SFT techniques *can* be implemented in individual sessions, they are not specifically developed for this type of session and consequently may require a higher level of adjustment by the therapist when delivered. For example, in individual sessions interventions related to family communication and attachment are adapted to explore the individual’s perception of family dynamics, rather than direct family interactions. A third factor is that session composition in routine care settings is not always an active clinical decision, but rather a result of impromptu family circumstances (i.e., some members are invited but refuse or cannot attend the session). Therapists may therefore need to adapt interventions originally planned for conjoint sessions while maintaining a systemic focus. These adaptations may make systemic interventions less readily identifiable to observers and contribute to differences between therapist and observer ratings. In summary, systemic interventions are fundamentally relational rather than behavioral. In conjoint sessions, these relational processes are externally observable because interaction occurs in real time, whereas in individual sessions they are often more symbolic, involving the exploration of internalized relationships. These processes remain systemic in intent but are less behaviorally explicit, making them more difficult for external observers to identify and more susceptible to differences between therapist intention and observer coding.

Finally, as expected, therapists delivered SFT techniques more frequently and extensively in conjoint sessions compared to individual sessions. Therapists did not routinely use the full set of SFT interventions in individual sessions, instead relying on only one or two techniques per session. The most often used are *Family communication and attachment* and *Parental monitoring and family rules*. Both these techniques often include coaching the individual on how to observe, reflect, and respond adaptively to family members. They can be used to promote self-awareness and help individuals develop more effective responses to family interactions, so they are easier to use when working with only one family member present than other techniques more focused on interactional change or relational reframing as *Core relational family themes*. These findings suggest that session composition influences not only the accuracy of therapist self-report but also the opportunities available to implement different types of systemic interventions.

## Strengths and limitations

The main strength of this study is its high ecological validity. It was conducted in a CMHC during routine care with families from diverse backgrounds, and with adolescents presenting with a diverse array of psychiatric diagnoses. Furthermore, therapists were trained in an ecological-systemic model of therapy that allowed them to understand and conceptualize cases from a systemic lens while retaining the flexibility to select interventions based on clinical need rather than following a manualized interventions. The study’s limitations pertain to the sample. Data were collected from a single clinic with a relatively small group of therapists who volunteered to participate and thus were not randomly selected. In addition, therapists selected which sessions to record and submit to the study, introducing the possibility of selection bias if sessions perceived as more successful or more representative of their practice were preferentially recorded. Finally, this study represents a secondary analysis of an existing observational dataset, and replication in additional community settings would strengthen the generalizability of these findings.

## Clinical implications

Therapists’ ability to provide reliable and accurate self-report on interventions they are delivering in session is critical to the advancement of psychotherapy research and practice, as self-report constitutes a key source of information about what occurs during the therapeutic process. Accurate self-assessment helps therapists and supervisors to identify opportunities for professional development, refine clinical practice, and guide supervision and training. Our findings indicate that, even without manualized protocols, family therapists can reliably and accurately report their overall use of SFT interventions in routine sessions, especially in conjoint sessions. However, therapists are less accurate and tend to overestimate the extent to which they are utilizing SFT interventions in individual sessions. This has the potential to influence clinical supervision, fidelity monitoring and even the clinical process. For example, therapists may put less effort into implementing interventions during future sessions under the false premise that the interventions are not effective, thereby decreasing therapists’ confidence in the effectiveness of the model (Hurlburt et al., 2010; Norcross and Lambert, 2018; Lambert and Harmon, 2018).

These findings carry important implications for supervision, quality assurance, and outcomes monitoring. Fidelity self-assessment is a very valuable supervision tool to understand opportunities for therapist professional development and thereby informs clinical training, so many clinics rely on therapist self-report to determine whether they are effectively providing systemic care. However, supervisors who rely on therapist progress notes may therefore be at risk of overestimating how much relational work actually happens in individual sessions, potentially missing opportunities to provide therapists with needed reflection and supervision. Moreover, these results indicate that supervisors who depend on therapist progress notes may overestimate in particular the relational work in individual sessions, suggesting that individual family therapy practice needs more oversight. These findings suggest that clinics and training programs may benefit from incorporating periodic observational coding or structured fidelity checks—especially for individual sessions—to complement therapist self-report and ensure an accurate understanding of clinical practice. In addition, implementation efforts that rely solely on therapist documentation may underestimate training needs, highlighting the importance of multi-method fidelity assessment to support therapists’ skill development and maintain the integrity of systemic family therapy models,

Future research should continue examining the clinical characteristics of specific systemic techniques, including their therapeutic goals, behavioral manifestations, and relational context, to better understand why some interventions are more consistently recognized than others. Such work may help us better understand how session composition affects the reliability and accuracy of therapist self-report, and what can be done to correct this therapist’s bias.

## Data Availability

The original contributions presented in the study are included in the article/supplementary material, further inquiries can be directed to the corresponding author/s.
